# Health Risk Assessment of Inhalable Particulate Matter in Beijing Based on the Thermal Environment

**DOI:** 10.3390/ijerph111212368

**Published:** 2014-11-28

**Authors:** Lin-Yu Xu, Hao Yin, Xiao-Dong Xie

**Affiliations:** State Key Joint Laboratory of Environmental Simulation and Pollution Control, School of Environment, Beijing Normal University, No. 19, Xinjiekouwai Street, Haidian District, Beijing 100875, China; E-Mails: yinhao@mail.bnu.edu.cn (H.Y.); xxd-830@163.com (X.-D.X.)

**Keywords:** PM_10_, urban heat island (UHI), remote sensing, health risk

## Abstract

Inhalable particulate matter (PM_10_) is a primary air pollutant closely related to public health, and an especially serious problem in urban areas. The urban heat island (UHI) effect has made the urban PM_10_ pollution situation more complex and severe. In this study, we established a health risk assessment system utilizing an epidemiological method taking the thermal environment effects into consideration. We utilized a remote sensing method to retrieve the PM_10_ concentration, UHI, Normalized Difference Vegetation Index (NDVI), and Normalized Difference Water Index (NDWI). With the correlation between difference vegetation index (DVI) and PM_10_ concentration, we utilized the established model between PM_10_ and thermal environmental indicators to evaluate the PM_10_ health risks based on the epidemiological study. Additionally, with the regulation of UHI, NDVI and NDWI, we aimed at regulating the PM_10_ health risks and thermal environment simultaneously. This study attempted to accomplish concurrent thermal environment regulation and elimination of PM_10_ health risks through control of UHI intensity. The results indicate that urban Beijing has a higher PM_10_ health risk than rural areas; PM_10_ health risk based on the thermal environment is 1.145, which is similar to the health risk calculated (1.144) from the PM_10_ concentration inversion; according to the regulation results, regulation of UHI and NDVI is effective and helpful for mitigation of PM_10_ health risk in functional zones.

## 1. Introduction

### 1.1. Background

Owing to the continuous development of the social economy and industrialization in China, urban regions are facing numerous environmental pollution problems, among which air pollution has become one of the most common. This is especially true for inhalable particulate matter (PM_10_), which represents a primary air pollutant that is detrimental to human health and has therefore received great attention from urban residents and governments. According to “The Key Environmental Air Quality Protection Cities in The First Half Year of 2012” [1] data published by the Chinese Ministry of Environmental Protection, the average PM_10_ concentration of 113 key environmental protection cities is 0.086 mg/m^3^, which exceeds the new air quality secondary standard (0.070 mg/m^3^) by 22.86%. Additionally, more than half of the cities in China, most of which are in northern China, did not pass the standards.

As a typical northern China city, Beijing is facing a serious problem of inhalable particulate matter pollution owing to increasing growth, construction, industrial production and the car population, coupled with the impact of external dust and specific climatic conditions. According to the Beijing Municipal Environmental Protection Bureau Beijing City 2011 Environmental Status Bulletin, the annual average concentration of PM_10_ is 0.121 mg/m^3^ in Beijing, which exceeds the new secondary standard by 72.86% and is 32.97% higher than the average concentration of the 113 key environmental protection cities in China [2].

With the recent increase in urbanization and the continuous expansion of city sizes, urban thermal environments are undergoing profound changes. As a result of this phenomenon, the strength and range of the heat island effect is expanding. In Beijing, climatic warming has been occurring at a rate of about 0.48 °C/decade during the last few decades (1977–2006) based on monitoring at 18 stations [3].

### 1.2. Study Review

When conducting health risk assessments most researchers reference the United States National Academy of Sciences (NAS) methodology, which mainly consists of four steps: hazard identification, dose response assessment, exposure assessment and risk characterization. Many studies have focused on toxic and harmful substances in inhalable particles health risk evaluation, including polycyclic aromatic hydrocarbons (PAH) [4] and other inorganic matter [5] and heavy metals [6]. Some researchers use epidemiological studies of PM_10_ health impacts as references, such as the relationship between exposure and response, to elucidate the relationship between the pollution level of inhalable particles and human health effects [7,8,9,10,11].

Under the effects of urban heat islands, urban areas suffer increasingly frequent extreme climatic events, such as heavy rain and heat waves. Additionally, air pollution in metropolitan areas is generally more serious, and has greater potential to affect human health and the ecological environment. These urban heat island (UHI) effects lead to changes in air quality [12] and increased concentrations of ozone [13] and fine particulate matter (PM_2.5_) or haze [14]. Studies have shown that there is a correlation between urban heat island intensity and the concentration distribution of inhalable particles [15,16,17]. In 1968, researchers found that the winds produced by urban heat island effects tend to sharpen pollution gradients between urban and rural areas [18]. One study in Paris indicated that UHI had an important impact on the primary and secondary regional pollutants [19]. Agarwal and Tandon in their study pointed out that the mesoscale wind produced by urban heat island help the pollutants to circulate and move in upward direction, thus making the problem of air pollution more severe in urban areas [20]. The poor air quality was associated with the greater frequency of a more intense UHI effect during the summer time, which was pronounced during the nighttime than the daytime [21]. Urban heat island can directly affect health because high temperatures place an added stress on human physiology [22]. Researches showed that excessive exposure to high heat was associated with increased rates of heat stress, heat stroke, and premature death [23]. The UHI effect could enhance health risks leading to higher mortality rates in cities compared to rural areas [24]. Moreover, the health risks associated with inhalable particulate matter are greatly influenced by the concentration, making it necessary to focus on the effects of UHI on the health risks of inhalable particulate matter.

Although many studies have been conducted to assess the health risk associated with inhalable particulate matter, few have investigated the regulation of inhalable particulate matter. Lichtenberg and Zilberman reported that an efficient health risk regulation model should be practical and useful for decision makers [17]. A range of health, safety, and environmental risk regulations have been implemented in both Europe and the United States during the last five decades [25], but these have mainly focused on certain toxic chemicals or hazardous materials [26]. The regulation is mostly conducted by the government and expressed as laws or through the political system, which seems to have powerful executive force. Toxicity studies have generally indicated that health risk regulation should first require an in-depth examination of the nature of the toxic risk problems themselves [27]. Accordingly, in a study of inhalable particles, health risk should be based on reasonable and accurate health risk analysis. Since no effective PM_10_ health risk regulation based on urban heat island effect has been established to date, the double-way regulation method established in this study is meaningful for urban environmental management.

Based on studies conducted in recent decades, it is essential to combine urban heat island effects with any PM_10_ health risk analysis system, which can be utilized for UHI effect mitigation and inhalable particulate matter reduction at the same time to promote urban sustainable development.

## 2. Methodology

In this study, we established a PM_10_ health risk assessment system based on the urban heat island effect. We utilized an established PM_10_ concentration-thermal environment model to integrate PM_10_ health risk assessment with urban heat island effect in different functional zones of Beijing. Comparisons between monitoring PM_10_ concentration/health risk and results based on thermal environment were made to make sure the model accuracy. Additionally, we adjusted the thermal environment indicators to regulate the health risk results in order to decrease the health risks and control the UHI effect simultaneously.

### 2.1. Study Area

Beijing is the capital of China, and one of the most populous cities in the world. The western, northern and northeastern portions of Beijing are surrounded by mountains, while the southeast is bordered by plains. The unique topography and climatic conditions of Beijing further aggravate the inhalable particulate matter pollution in the city by preventing particulate diffusion.

**Figure 1:**
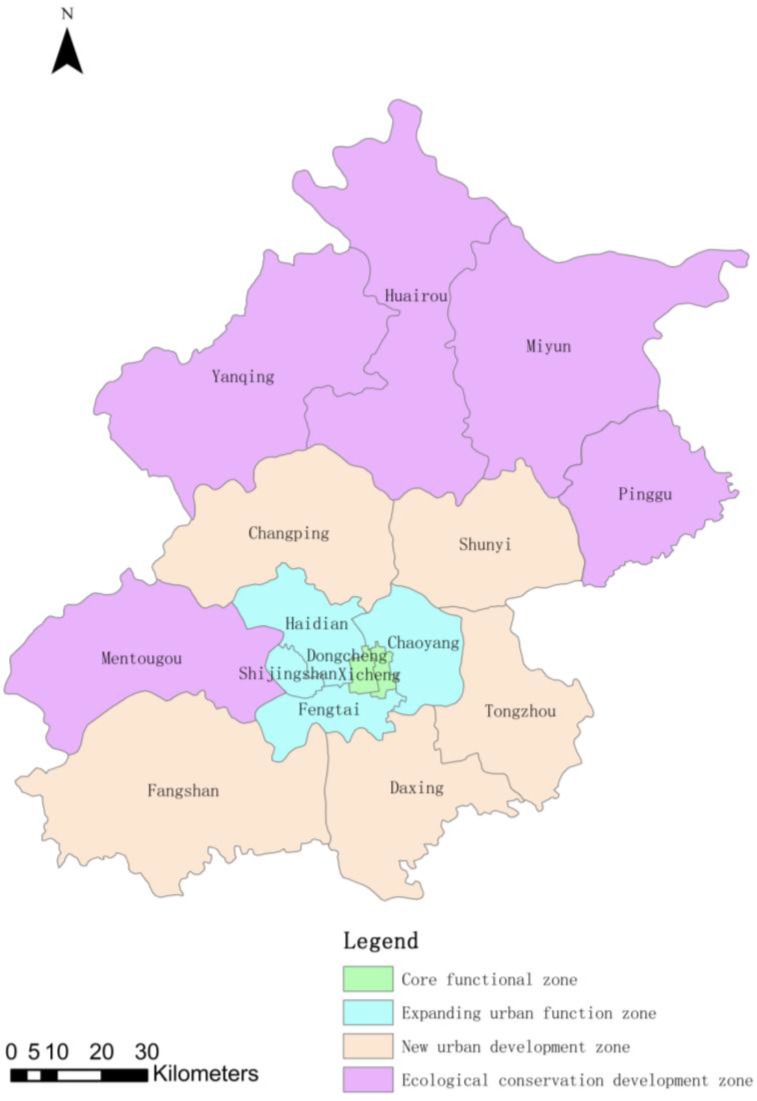
Study area and functional regionalization distribution.

To promote sustainable economic and social development and optimize the overall function of the capital, Beijing has implemented a functional plan pertaining to its 14 urban and suburban districts and two rural counties (Figure 1). In this plan, districts are divided into four functional regions: core functional zone (Dongcheng, Xicheng districts), new urban expanding urban functional zone (Chaoyang, Fengtai, Shijingshan and Haidian districts), new urban development zone (Fangshan, Tongzhou, Shunyi, Changping, and Daxing districts) and ecological conservation development zone (Mentougou, Huairou, and Pinggu districts and Miyun, Yanqing counties).

Because of the different functional zones with various population levels, structures of energy consumption and regional GDP, the PM_10_ health risk assessment of different functional zones in Beijing is more applicable than direct evaluation of the entire city for urban atmospheric environmental management and planning; therefore, this study focused on functional regions and illustrates the reasons for high risk level in certain districts.

### 2.2. Remote Sensing Data

Landsat 5 Thematic Mapper (TM) data were developed by the National Aeronautics and Space Administration (NASA). The satellite, launched in March 1984 [28], is one of the longest running and widely used satellites today. The repeat interval of Landsat 5 is 16 days, which means that we can obtain data from 2–3 TM images in a month. As a result of this, it is difficult to obtain high quality data in one season. In this study, we utilized the Landsat 5 TM image retrieval method to estimate the PM_10_ concentration. At present, TM images are available from The Institute of Remote Sensing and Digital Earth (RADI), Chinese Academy of Sciences (CAS) [29].

Higher particle concentration during the heating period in Beijing is due to the coal-burning infrastructure, and always shows a very high incidence of epidemic disease during the spring season; consequently, health risks associated with inhalable particulate matter are more serious during this period. Therefore, it is dramatically imperative to pay attention to this season and mitigate the high health risks due to the PM_10_ pollution. Based on this consideration, the TM image of Beijing on 14 March 2009 was acquired on a clear-sky day as the basic data for PM_10_ health risk analysis.

The inhalable particulate matter increase will cause the transmissivity of visible light and near infrared light to decrease, moreover, the transmissivity of near infrared light drops faster than that of the visible light [30]. Therefore, it is feasible to adopt the difference of visible light and near infrared light transmissivity with dual channel technology to establish the difference vegetation index (DVI). With the help of DVI index, we established a correlation between PM_10_ concentration and DVI index in Beijing. We also obtained the daily average concentration of inhalable particulate matter data from the Beijing environmental protection monitoring center [31] to establish the correlation between DVI and PM_10_ concentration.

### 2.3. PM_10_ Health Risk Assessment

In this study, we utilized PM_10_ remote sensing inversion and monitoring data to analyze heath risk based on an epidemiological study. Moreover, another health risk assessment was conducted based on thermal environment, which is meaningful for PM_10_ health risk control and management.

#### 2.3.1. PM_10_ Health Impact Identification

Inhalable particles cause various respiratory and cardiovascular diseases and increase the number of inpatients, outpatients and mortality [32,33,34,35]. Inhalable particulate matter health impacts are divided into three categories according to their degree of harm: death, including chronic death and acute death (referred to as all-cause mortality); disease, including asthma, chronic bronchitis and acute bronchitis; and hospitalization, including respiratory system disease in the hospital and cardiovascular hospitalization [36] (see Table 1).

**Table 1:** Exposure-response relationship coefficients of different diameters of PM_10_ (β_i_).

Hazard Level	Health Impact Types (i)	β_i_ (PM_10_)		Reference Information
		Average	95% Confidence Interval	
Death	All causes mortality	0.00038	(0.00035, 0.00042) [37]	Meta analysis based on Chinese studies, 2009
	Chronic mortality	0.00192 *	(0.000494, 0.00328) * [38]	Meta analysis based on Chinese studies, 2013
	Acute mortality	0.00026 *	(0.000124, 0.000403) * [38]	Meta analysis based on Chinese studies, 2013
Morbidity	Asthma	0.00190	(0.00145, 0.00235) [37]	Meta analysis based on Chinese studies, 2009
	Chronic bronchitis	0.00656 *	(0.00238, 0.01013) * [38]	Meta analysis based on Chinese studies, 2013
	Acute bronchitis	0.00550	(0.00189, 0.00911) [39]	Study in Pearl River Delta in China, 2006
Hospitalization	Respiratory system disease	0.00124	(0.00084, 0.00162) [39]	Study in Pearl River Delta in China, 2006
	Cardiovascular disease	0.00066	(0.00036, 0.00095) [39]	Study in Pearl River Delta in China, 2006

Notes: * indicates that the data were converted by PM_2.5_/PM_10_ = 0.65, all data were collected from recent studies.

#### 2.3.2. PM_10_ Exposure-Response Assessment

Epidemiological studies have revealed the correlation coefficient of the changes of some health effects caused by variations in inhalable particulate matter concentration, namely the exposure-response coefficient. The health impacts of inhalable particulate matter are closely related to the physical status of local residents and climate conditions, so exposure response relationship factors should be selected as references from domestic epidemiological studies whenever possible, and data from other areas should be considered when appropriate.

This study investigated the studies of exposure-response coefficients of PM_10_ in China; however, the dataset used for this analysis was incomplete. Most domestic epidemiological research includes analysis of health impacts and the exposure-response relationship of domestic PM_10_ and PM_2.5_ based on the meta-analysis method. Such analysis showed that the Pearl River Delta Region were subject to inhalable particulate matter pollution at levels that caused severe health impacts [37]. Additionally, the association between ambient air pollutants and increased hospital emergency room visits for cardiovascular diseases in Beijing, China were investigated [40]. Moreover, some studies have evaluated PM_2.5_ exposure-response relationship coefficients in some cities in China [9,38]. Recent studies showed that PM_2.5_/PM_10_ showed a certain proportion in Beijing. One study showed that the annual PM_2.5_/PM_10_ mass ratio was 0.71 in Beijing [41]. Another research showed that the PM_2.5_/PM_10_ ratios at the surface sites ranged from 37.5% to 85.1% with noticeably higher average values of 56.1%–66.5% at urban and elevated sites [42]. And long-term monitoring of PM_2.5_/PM_10_ concentration study pointed out that the proportion of PM_2.5_/PM_10_ was about 61.5% from 2001–2006 [43]. Therefore, based on the studies in China, we assume that the PM_2.5_/PM_10_ is 0.65 in general to obtain the exposure-response coefficients [37]. The exposure-response coefficients of relative health impacts are shown in Table 1.

#### 2.3.3. PM_10_ Health Risk Characterization

This study employed a relative risk model based on Poisson Regression [7,9,11], which is commonly used in epidemiological studies of air pollution to calculate the relative risk of inhalable particulates with certain health impacts. We then adopted the average relative risk of all health impacts to represent the health risk of inhalable particulate matter using the following equations:

(1) $${\text{TR}}_{\text{i}}=\frac{{\text{R}}_{\text{i}}}{{\text{R}}_{0\text{i}}}={\text{e}}^{{\text{β}}_{\text{i}}\times \left(\text{C}-{\text{C}}_{0}\right)}$$

(2) $$\text{TR}=\frac{1}{\text{n}}\overset{\text{n}}{\underset{\text{i}=1}{\sum }}{\text{TR}}_{\text{i}}$$

where, TR is the health risk of inhalable particulate matter; TR*_i_* is the relative risk caused by the *i*th health impact, i = 1,2,3,…,7 (see Table 1); R_i_ is the actual risk of the *i*th health impact; R_0i_ is the reference risk value of the *i*th health impact; β_i_ is the exposure-response coefficient; C is the actual concentration of inhalable particulate matter; C_0_ is the reference concentration in the risk assessment based on the average year guiding value of inhalable particulate matter set by the WHO, *i.e.*, PM_10_ is 20 μg/m^3^; *n* is the number of health impact types caused by inhalable particulate matter.

#### 2.3.4. PM_10_ Health Risk Assessment Based on Thermal Environment

In this study, we utilized infrared temperature to retrieval the surface temperature, and then obtained the UHI, NDVI, NDWI and DVI indicators according to the following equations.

UHI indicator calculation:

(3) $${\text{L}}_{\text{b}}={\text{L}}_{\text{min}}+\;\frac{{\text{L }}_{\text{max}}-{\text{L}}_{\text{min}}}{{\text{DN}}_{\text{max}}}\times \text{DN}$$

where L_b_ means the radiation brightness; L _max_ and L _min_ refer to the maximum and minimum radiation intensities; DN represents the gray value of band 6; L _min_ = 0.1238 mW·cm^−2^·sr^−1^·μm^−1^, L _max_ = 1.56 mW·cm^−2^·sr^−1^·μm^−1^, and DN _max_ = 255.

(4) $${\text{T}}_{\text{b}}=\frac{{\text{K}}_{2}}{\text{ln}\left({\text{K}}_{1}/{\text{L}}_{\text{b}}+1\right)}$$

where T_b_ brightness temperature; K_1_ and K_2_ are constants (K_1_ = 60.776 mW·cm^−2^·sr^−1^·μm^−1^, K_2_ = 1260.56 K).

(5) $${\text{T}}_{\text{R}}=\;\frac{{\text{T}}_{\text{i}}-{\text{T}}_{\text{a}}}{{\text{T}}_{\text{a}}}$$

where T_R_ is the relative brightness temperature, which represents the UHI index in this study; T_i_ refers to certain point (i) brightness temperature (T_b_), and T_a_ means the average brightness temperature.

NDVI reflects the vegetation coverage and growth state from space [44]. NDVI indicator calculation:

(6) $$\text{NDVI}=\;\frac{\text{NIR}-\text{R}}{\text{NIR}+\text{R}}$$

where R and NIR represent red (λ~0.6 μm) and near infrared (λ~0.8 μm) reflectivity.

NDWI refers to the differences of water surface content [45]. NDWI indicator calculation:

(7) $$\text{NDWI}=\frac{\text{NIR}-\text{MIR}}{\text{NIR}+\text{MIR}}$$

where NIR and MIR represent the near infrared (λ~0.8 μm) and middle infrared (λ~1.65 μm) reflectivity, respectively.

DVI indicator calculation:

(8) DVI=NIR−R

where R and NIR represent red (λ~0.6 μm) and near infrared (NIR) (λ~0.8 μm) reflectivity.

The PM_10_ health risk assessment model is considered to adopt the PM_10_ concentration equations (Equation (9)) generated by Xu *et al.* (2013), which is based on the correlation between the PM_10_ concentration and thermal environmental indicators (UHI, NDVI, and NDWI) [46]. We then utilized the concentration formula to calculate PM_10_ health risk (Equation (10)) with epidemiological method from Equation (1):

(9) $$\{\begin{array}{c} \text{Core}\;\text{functional}\;\text{zone}:\text{y}\;=\;-4.885{\text{x}}_{1}-2.370\\ \text{Expanding}\;\text{urban}\;\text{functional}\;\text{zone}:\text{y}=\;-1.391{\text{x}}_{1}+82.246{\text{x}}_{2}+0.164{\text{x}}_{3}+0.132\\ \text{New}\;\text{urban}\;\text{development}\;\text{zone}:\text{y}=0.917{\text{x}}_{1}+90.329{\text{x}}_{2}+0.0215\\ \text{Ecological}\;\text{conservation}\;\text{development}\;\text{zone}:\text{y}=0.0401{\text{x}}_{1}+62.470{\text{x}}_{2}+0.620 \end{array}$$

(10) $${\text{TR}}_{\text{i}}={\text{e}}^{{\text{β}}_{\text{i}}\times \left(\text{F}(\text{DVI})-{\text{C}}_{0}\right)}={\text{e}}^{{\text{β}}_{\text{i}}\times \left(\text{F}\left(\text{f}\left(\text{UHI},\text{ NDVI},\text{ NDWI}\right)\right)-{\text{C}}_{0}\right)}$$

where, f (UHI,NDVI,NDWI) refers to the PM_10_ concentration calculation formulas in Equation (7); y is the value of DVI (Difference vegetation index) in Equation (8); x_1_, x_2_, and x_3_ represent UHI, NDVI and NDWI respectively Equations (3)–(7). Other parameters are explained in Equation (1).

### 2.4. PM_10_ Health Risk Regulation

This study utilized the PM_10_ health risk analysis model combined with thermal environment indicators to regulate PM_10_ health risk by adjusting the UHI intensity. To illustrate the PM_10_ health risk regulation effect, we set three scenarios by regulating UHI, NDVI and NDWI to illustrate which indicator influences the PM_10_ health risk most significantly. Scenario 1 is the UHI regulation, in which we adjust the UHI by 0.1, and then analyzed the PM_10_ health risk spatial changes in different districts and counties of Beijing utilizing the Zonal Statistics function in ArcGIS. In Scenario 2, we regulated UHI and NDVI together to figure out NDVI influences on the PM_10_ health risk. In Scenario 3, we added NDWI indicators into Scenario 2, and then compared the three scenarios and analyze the differences among them.

## 3. Results and Discussion

### 3.1. Remote Sensing Inversion of PM_10_ Concentration in Beijing

**Figure 2:**
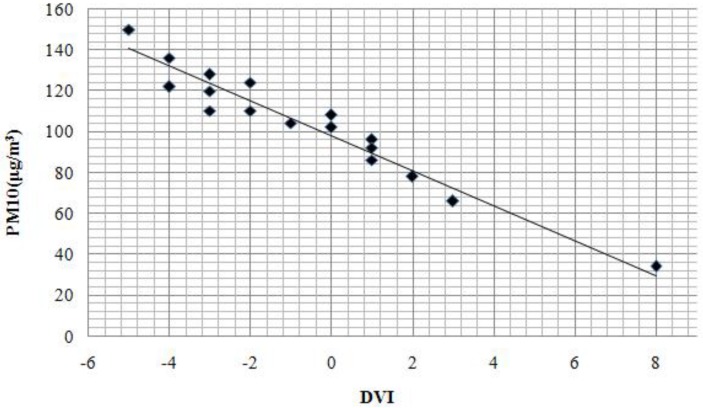
Relationship between DVI and PM_10_ concentration in March 2009.

The DVI index is built due to the different influence of inhalable particle pollutants on the transmissivity of the visible channel and near infrared channel of the NOAA satellite [30]. The DVI (difference vegetation index) was used to determine the inverse spatial distribution of inhalable particulate matter. Recent studies indicate that there is a linear correlation between DVI and PM_10_ [30,47]. We used the PM_10_ synchronous monitoring data collected from 17 Beijing ground stations taken when the Landsat Satellite transited Beijing (see Figure 2). The DVI values were then extracted according to the geographic coordinates of the stations. To diminish impacts on the final results due to location errors, the average DVI values of 3 × 3 pixels around the monitoring station were used. SPSS software analysis of the linear correlation of the monitoring data of PM_10_ and DVI values generated a correlation coefficient of −0.9683. The linear regression equation describing the relationship between PM_10_ concentration and DVI was then established and the following regression equation of the PM_10_ concentration and the DVI values based on the TM images in 2009 was generated (Equation (11)):

(11) $$\text{y}\;=\;-8.533\text{x}\;+\;97.94{\;}^{2}\;=0.937$$

where y is the concentration of PM_10_ (μg/m^3^) and x is the DVI.

**Figure 3:**
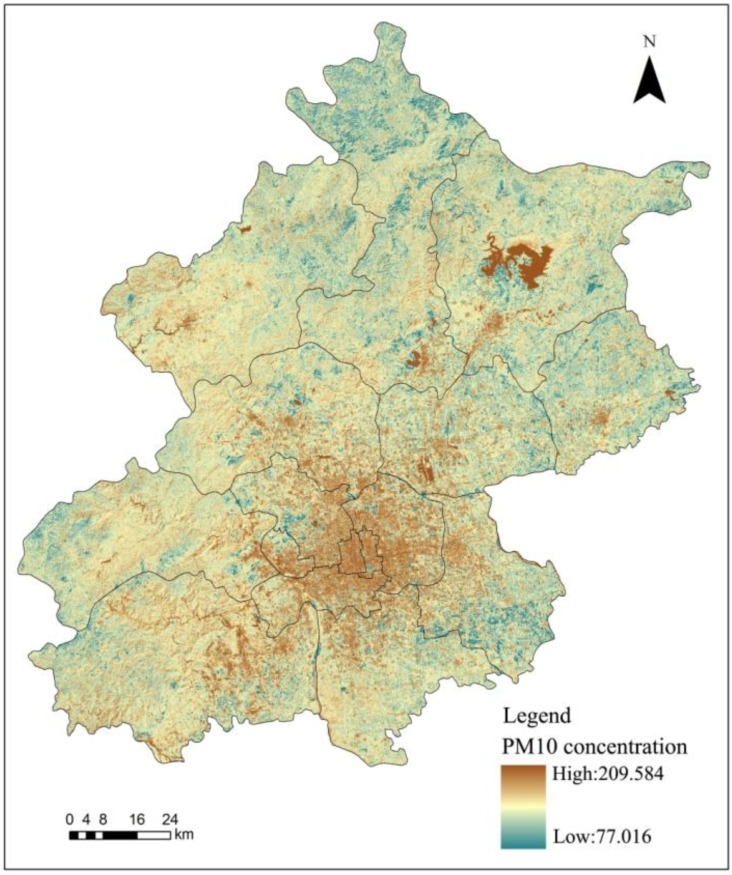
PM_10_ TM image inversion results in March 2009.

The Spatial Analyst tool in ArcGIS was used to establish the inverse model based on the regression equation to give the inverse PM_10_ spatial distribution for Beijing in 2009 (Figure 3). Due to the fact that water surface has a very low reflection; therefore, the DVI values are influenced by this and has a much lower values than the other areas. Thus it is illustrated clear that large water surface areas all have a relative high PM_10_ inversion concentration. Therefore, the PM_10_ concentration reversion results of Miyun reservoir and other large water surface areas should not be taken into consideration. To make the study results accurate, we have deleted the PM_10_ concentration of the water surface areas in the study area. The PM_10_ concentration retrieval method is not suitable for the water surface; therefore, the Miyun reservoir PM_10_ concentration reversion results could not be taken into consideration. Except for some unique areas such as the Miyun reservoir, the spatial distribution of the inverse PM_10_ concentration from the TM images in 2009 were generally in line with the spatial distribution characteristics of inhalable particulate matter in Beijing, with PM_10_ concentrations in urban areas being larger than in suburbs and southwestern PM_10_ concentrations being larger than those in the northeast. The statistical analysis function also revealed that the average PM_10_ concentration in Beijing is 81.507 μg/m^3^, while the west area of the city had the largest PM_10_ concentration of 125.958 μg/m^3^, and that of the Huairou district had the lowest PM_10_ concentration of 66.464 μg/m^3^.

Validation samples were selected at random based on the regression equations for accuracy verification using Equation (12) to acquire the results shown in Table 2. We excluded the largest and smallest error rates during statistical analysis to obtain reliable results. The results of the 2009 PM10 inversion of the TM image had a smaller error and higher precision. The final average error rate was 8.44%, indicating that the error of the PM10 concentration inversion results in 2009 was relatively small and authentic:

(12) $$ER=\frac{\left|C_{i}-C_{j}\right|}{C_{j}}$$

where ER is the error rate of the PM_10_ concentration based on thermal environment, *C_i_* is the value of the PM_10_ concentration based on thermal environment, *C_j_* is the actual value of the PM_10_ concentration.

**Table 2:** Accuracy verification results of PM_10_ TM image inversion in March 2009.

Sample Serial Number	1	2	3	4	5	6	7
Error rate (%)	3.98	16.68	5.66	14.39	0.05	11.76	6.41
Average error rate (%)	8.44						

### 3.2. PM_10_ Health Risk Assessment in Beijing

**Figure 4:**
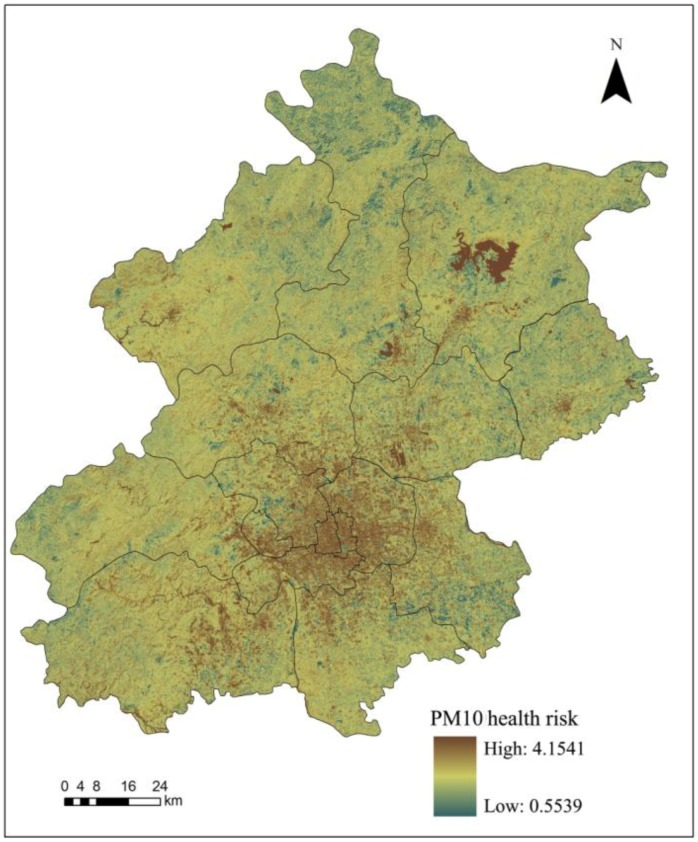
PM_10_ health risk assessment results in Beijing in March 2009.

According to the inhalable particulate matter risk assessment method, we used the remote sensing inversion of PM_10_ spatial distribution to calculate the corresponding relative risk (TR_i_) to the certain health impact (i) of inhalable particulate matter (Equation (1)), after which we calculated the inhalable particulate matter health risk assessment (TR) according to Equation (2). The calculation results are shown in Figure 4.

The spatial distribution of the PM_10_ health risk assessment results is basically the same as the inhalable particulate matter spatial distribution in Beijing in 2009; with a higher health risk in urban areas than rural areas and southwest regions than northeast regions (Figure 4). Additionally; we excluded specific regions such as the Miyun reservoir and obtained an average health risk value of 1.144. Statistical analysis revealed a descending health risk in central areas of the city; including the Dongcheng; Xicheng; Chaoyang; Fengtai; Shijingshan and Haidian districts; as well as in the new urban development zone; which comprises the Fangshan; Changping; Tongzhou; Shunyi and Daxing districts. However; in the ecological conservation development zone; the PM_10_ health risk was increasing from the Pinggu; Mentougou; and Huairou districts to Miyun and Yanqing counties.

When Miyun reservoir and other special areas are excluded, the health risk associated with PM_10_ in Beijing was 1.144. The results indicated that health risks associated with inhalable particulate matter occurred in the following order: Dongcheng > Xicheng > Chaoyang > Fengtai > Shijingshan > Haidian districts, as well as: Fangshan > Changping > Tongzhou > Shunyi > Daxing districts in the new urban development area and Pinggu > Mentougou > Huairou > Miyun > Yanqing in the ecological conservation area.

### 3.3. PM_10_ Health Risk Assessment Based on Thermal Environment in Beijing

We calculated the average UHI, NDVI and NDWI and utilized these indicators to compute the PM_10_ concentration values in different districts or counties in Beijing in March 2009 (Table 3). The calculation equations of UHI, NDVI and NDWI have been conducted and published by Xu *et al.* [46].

**Table 3:** UHI, NDVI and NDWI and PM_10_ concentration of different districts/counties in March 2009.

Function Zone	Districts/Counties	UHI	NDVI	NDWI	PM_10_
Core functional zone	Dongcheng	0.0949	−0.0340	2.1858	122.1179
	Xicheng	0.1032	−0.0479	2.26298	122.4633
Expanding urban functional zone	Chaoyang	0.1347	−0.0110	1.82502	103.6067
	Fengtai	0.2059	−0.0087	1.63885	103.0618
	Shijingshan	0.1738	−0.0027	1.51558	98.6612
	Haidian	0.1295	0.0075	1.46408	91.0722
New urban development zone	Fangshan	0.0821	0.0142	1.88004	86.1678
	Tongzhou	0.1630	0.0240	1.64704	77.9883
	Shunyi	0.1159	0.0233	1.04961	78.8774
	Changping	0.1718	0.0201	1.16889	80.9161
	Daxing	0.1980	0.0169	1.12659	83.1933
Ecological conservation development zone	Mentougou	−0.0294	0.0246	1.59843	79.5299
	Huairou	0.0314	0.0482	1.87591	66.9317
	Pinggu	−0.2141	0.0423	2.11136	70.1956
	Miyun	−0.1543	0.0325	1.79686	75.3956
	Yanqing	−0.1562	0.0307	1.75038	76.3468

Note: “PM_10_” represents the average PM_10_ concentration (μg/m^3^) in different districts or counties.

According to the PM_10_ concentration calculated based on the thermal environment, we obtained the health risks of Beijing in March 2009. The results indicated that the health risk results based on thermal environment were similar to the previous assessment results calculated from PM_10_ remote sensing inversion, which was with an average variance ratio of 0.38% and the largest variance ratio being 1.05% (Table 4). These findings indicate that the PM_10_ health risk assessment method based on thermal environment can present PM_10_ health risks in the region with relatively good precision.

**Figure 5:**
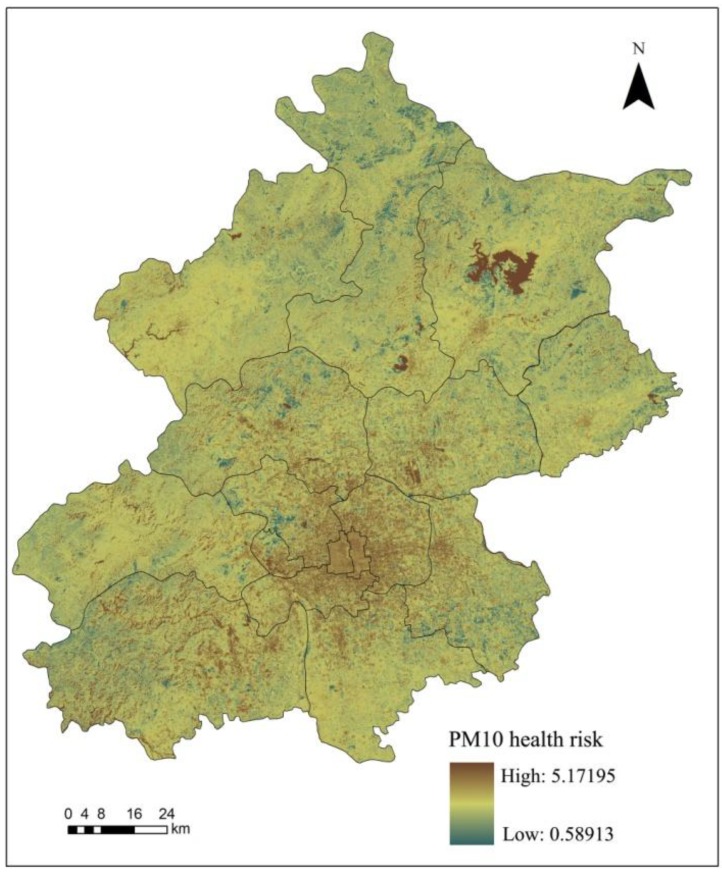
PM_10_ health risks based on the thermal environment in Beijing in March 2009.

To compare the PM_10_ risk assessment results based on thermal environment with the previous results in part 3.3, the Zonal Statistics function in the ArcGIS software was used to analyze the statistical results. It was indicated that the analysis of PM_10_ health risks based on thermal environment was roughly the same as the PM_10_ spatial distribution in Beijing (Figure 5). The assessment results showed that the health risk of urban areas was higher than the health risk of rural areas and the southwest region had a higher risk than northeast regions. After excluding some unique regions such as the Miyun reservoir, we obtained the average health risk associated with PM_10_ of 1.145.

**Table 4:** PM_10_ health risk assessment results comparison in Beijing in March 2009.

Function Zone	District/County	PM_10_ Health Risk Assessment		
		Results 1 (TR_a_) CI (95%)	Results 2 (TR_b_) CI (95%)	Variance Ratio (%)
Core functional zone	Dongcheng	1.2876 (1.1052, 1.5196)	1.3012 (1.1094, 1.5482)	1.0593
	Xicheng	1.3157 (1.1139, 1.5789)	1.3025 (1.1098, 1.5509)	1.0025
Expanding urban functional zone	Chaoyang	1.2355 (1.0883, 1.4138)	1.2351 (1.0882, 1.4131)	0.0291
	Fengtai	1.2351 (1.0882, 1.4130)	1.2333 (1.0876, 1.4094)	0.1451
	Shijingshan	1.2254 (1.0850, 1.3940)	1.2185 (1.0827, 1.3803)	0.5707
	Haidian	1.1962 (1.0751, 1.3374)	1.1937 (1.0742, 1.3326)	0.2104
New urban development zone	Fangshan	1.1754 (1.0679, 1.2982)	1.1781 (1.0599, 1.2568)	0.2273
	Tongzhou	1.1469 (1.0577, 1.2457)	1.1530 (1.0609, 1.2617)	0.5301
	Shunyi	1.1514 (1.0594, 1.2539)	1.1557 (1.0631, 1.2731)	0.3682
	Changping	1.1694 (1.0658, 1.2870)	1.1619 (1.0656, 1.2860)	0.6436
	Daxing	1.1657 (1.0644,1.2870)	1.1689 (1.0616, 1.2653)	0.2727
Ecological conservation development zone	Mentougou	1.1583 (1.0618, 1.2666)	1.1576 (1.0688, 1.3032)	0.0588
	Huairou	1.1192 (1.0476, 1.1962)	1.1205 (1.0481, 1.1986)	0.1192
	Pinggu	1.1248 (1.0496, 1.2061)	1.1299 (1.0515, 1.2153)	0.4590
	Miyun	1.1429 (1.0563, 1.2385)	1.1452 (1.0571, 1.2427)	0.2026
	Yanqing	1.1509 (1.0592, 1.2530)	1.1481 (1.0581, 1.2478)	0.2499
Average value		1.1875	1.1877	0.3843

Notes: “Results 1” means the health risk results calculated based on the PM10 inversion of remote sensing, “Results 2” represents the health risk assessment results based on thermal environment. Variance ratio (%) **=** |TR_a_ − TR_b_|/TR_a_ × 100.

### 3.4. PM_10_ Health Risk Regulation in Beijing

There is a certain relationship between UHI and NDVI, -which means the increase of NDVI may cause the temperature mitigation or UHI intensity reduction. Due to the fact that complex processes are involved in determining the cooling effect of vegetation on daytime air and surface temperature [32], there is no authentic correlation of the two indicators obtained from recent studies.

There is also no accurate relationship between UHI intensity and NDWI. In this study, we assume that UHI, NDVI and NDWI indicators are relatively independent indicators to set three regulation scenarios:

*Scenario 1:* UHI regulation. To promote the urban atmospheric environment management, in this study, we decreased UHI indicator for the value of 0.1 and analyzed the variation of PM_10_ health risk in Beijing in March 2009. Results showed that the core functional zone and expanding urban functional zone were found to have positive regulation effects, with average regulation effects of 0.0152 and 0.0069 (Table 5). Additionally, after reducing UHI indicator of 0.1, the inhalable particulate matter health risk decreased by 1.52% and 0.69% in the two zones. Conversely, the new urban development zone and ecological conservation development zone regulation effects were negative, that was, and the reduction of UHI intensity value leads to the increase of PM_10_ health risk.

From the health risk assessment results (Table 5), it is claimed that the health risks in Core functional zone (average 1.3016) and Expanding urban functional zone (average 1.2230) are higher than the New urban development zone (average 1.1618) and Ecological conservation development zone (average 1.1392). Therefore, the UHI regulation could be more effective with higher health risks, whereas, the health risk regulation could be adverse with lower health risk in certain circumstances. It is illustrated that UHI regulation can be effective in relative high-risk areas while can be adverse in some low health risk regions.

**Table 5:** Beijing PM_10_ health risk regulation results analysis (UHI-0.1).

Function Zone	District/County	Assessment Results	Regulation Results	Regulation Effects
Core functional zone	Dongcheng	1.2876	1.2858	0.0018
	Xicheng	1.3157	1.2870	0.0286
	Average	1.3016	1.2864	0.0152
Expanding urban functional zone	Chaoyang	1.2355	1.2311	0.0044
	Fengtai	1.2351	1.2292	0.0058
	Shijingshan	1.2254	1.2145	0.0109
	Haidian	1.1962	1.1899	0.0063
	Average	1.2230	1.2162	0.0069
New urban development zone	Fangshan	1.1754	1.1806	−0.0051
	Tongzhou	1.1469	1.1553	−0.0084
	Shunyi	1.1514	1.1580	−0.0066
	Changping	1.1694	1.1643	0.0051
	Daxing	1.1657	1.1713	−0.0056
	Average	1.1618	1.1659	−0.0041
Ecological conservation development zone	Mentougou	1.1583	1.1577	0.0006
	Huairou	1.1192	1.1206	−0.0014
	Pinggu	1.1248	1.1300	−0.0053
	Miyun	1.1429	1.1453	−0.0024
	Yanqing	1.1509	1.1482	0.0028
	Average	1.1392	1.1404	−0.0012

Note: regulation effect = assessment result-regulation result.

*Scenario 2*: UHI and NDVI regulation. Based on *Scenario 1*, we increased the NDVI indicator by 0.1 to figure out the variation of regulation effects. Table 6 shows the regulation effects after the adjustment of UHI and NDVI in different functional zones.

It is obvious that *Scenario 2* has better regulation effects than *Scenario 1*, and in expanding urban functional zone, new urban development zone and ecological conservation development zone, the health risks decline by 20.48%, 19.48% and 13.82% respectively, while the health risk is the consistent with *Scenario1* in core functional zone.

*Scenario 3*: UHI, NDVI and NDWI regulation. In this scenario, we decreased UHI by 0.1 and increased NDVI and NDWI by 0.1 respectively to analyze the health risk in different districts or counties in Beijing. The calculation results compared with the health risk assessment results are listed in Table 7. As the results illustrated, only in expanding urban functional zone there is a little improvement (0.0003) in regulation effects, while the other zones have the same results compared with Scenario 2 (Table 7). The results may be due to the fact that the NDVI and NDWI show little correlation with the DVI indicator in core functional zone (Equation (3)). Therefore, the increase of NDVI and NDWI does not reduce the health risk of core functional zone obviously. Moreover, NDWI is directly correlated with DVI in expanding urban functional zone only, as a result of this, the regulation of NDWI influences little on the PM10 health risks in the other functional zones.

**Table 6:** Beijing PM_10_ health risk regulation results analysis (UHI-0.1, NDVI + 0.1).

Function Zone	District/County	Regulation Results	Regulation Effects
Core functional zone	Dongcheng	1.2858	0.0018
	Xicheng	1.2870	0.0286
	Average	1.2864	0.0152
Expanding urban functional zone	Chaoyang	1.0290	0.2065
	Fengtai	1.0276	0.2074
	Shijingshan	1.0171	0.2084
	Haidian	0.9993	0.1969
	Average	1.0183	0.2048
New urban development zone	Fangshan	0.9772	0.1982
	Tongzhou	0.9595	0.1874
	Shunyi	0.9614	0.1900
	Changping	0.9658	0.2036
	Daxing	0.9707	0.1950
	Average	0.9669	0.1948
Ecological conservation development zone	Mentougou	1.0146	0.1437
	Huairou	0.9856	0.1336
	Pinggu	0.9930	0.1318
	Miyun	1.0049	0.1380
	Yanqing	1.0071	0.1438
	Average	1.0010	0.1382

**Table 7:** Beijing PM_10_ health risk regulation results analysis (UHI-0.1, NDVI + 0.1, NDWI + 0.1).

Function Zone	District/County	Regulation Results	Regulation Effects
Core functional zone	Dongcheng	1.2858	0.0018
	Xicheng	1.2870	0.0286
	Average	1.2864	0.0152
Expanding urban functional zone	Chaoyang	1.0286	0.2068
	Fengtai	1.0273	0.2077
	Shijingshan	1.0167	0.2087
	Haidian	0.9990	0.1972
	Average	1.0179	0.2051
New urban development zone	Fangshan	0.9772	0.1982
	Tongzhou	0.9595	0.1874
	Shunyi	0.9614	0.1900
	Changping	0.9658	0.2036
	Daxing	0.9707	0.1950
	Average	0.9669	0.1948
Ecological conservation development zone	Mentougou	1.0146	0.1437
	Huairou	0.9856	0.1336
	Pinggu	0.9930	0.1318
	Miyun	1.0049	0.1380
	Yanqing	1.0071	0.1438
	Average	1.0010	0.1382

However, we must admit that the correlation equations show the main oriented correlation types, which means that NDVI and NDWI still influence the concentration of inhalable particulate matter in core functional zone. To achieve the goal of PM_10_ health risk mitigation of Beijing in March 2009, *Scenario 2* and *Scenario 3*, which can control the UHI effect and improve the vegetation coverage in urban areas are very acceptable and effective, although for environmental management and control, *Scenario 2* is more practicable than the other scenarios.

## 4. Conclusions

This study established a PM_10_ health risk assessment system based on the urban thermal environment utilizing the epidemiological method combined with remote sensing inversion and monitoring techniques to provide a proposal for urban inhalable particulate matter regulation and management. The PM_10_ health risk of Beijing showed two distribution aspects in March 2009; namely, PM_10_ health risk in urban areas was higher than in rural areas and the southwest than in the northeast portion of the city and different functional regions showed spatial variation. Utilizing the PM_10_ health risk assessment model based on the thermal environment, the PM_10_ health risk in Beijing was determined to be 1.145, which is close to the health risk assessment results (1.144) derived from the PM_10_ concentration inversion with remote sensing method. These findings illustrate that the PM_10_ health risk assessment system based on thermal environment is acceptable and meaningful for urban environment management as well as UHI effect and PM_10_ health risk control. According to the health risk regulation of UHI, NDVI and NDWI, it is very effective to control the UHI and NDVI indicators for urban PM_10_ health risk management. Therefore, for urban heat island effect control and PM_10_ mitigation, the regulation of the UHI and NDVI together is meaningful and useful. In this research, although have attempted to give general study conclusions at best, there are still some uncertainties that need to be considered The remote sensing data obtained in this study could be limited, while we obtained the TM image on a typical weather condition day, which could reflect the general health risk situation at certain extent. Moreover, the comprehensive health risk is based on the health endpoints selected in this study that may not cover all the health endpoints due to the PM_10_ pollution or have some overlap among them. Whereas, the health endpoints here are selected in three levels, which could be relative authentic and appropriate for the health risk assessment. As a whole, this study proposes a general solution to mitigate the urban heat island effect as well as the PM_10_ health risk in urban areas, which could give suggestions for urban management.
